# Targeting Ferroptosis in Parkinson’s: Repurposing Diabetes Drugs as a Promising Treatment

**DOI:** 10.3390/ijms26041516

**Published:** 2025-02-11

**Authors:** Carmen Duță, Corina Muscurel, Carmen Beatrice Dogaru, Irina Stoian

**Affiliations:** Department of Biochemistry, Carol Davila University of Medicine and Pharmacy, 050474 Bucharest, Romania; carmen.duta@umfcd.ro (C.D.); corina.muscurel@umfcd.ro (C.M.); irina.stoian@umfcd.ro (I.S.)

**Keywords:** Parkinson’s disease, type 2 diabetes medication, oxidative stress, ferroptosis

## Abstract

This review explores the promising potential of repurposing type 2 diabetes (T2D) medications for the treatment of Parkinson’s disease (PD), highlighting the shared pathophysiological mechanisms between these two age-related conditions, such as oxidative stress, mitochondrial dysfunction, and ferroptosis. The overlap suggests that existing diabetes drugs could target the common pathways involved in both conditions. Specifically, the review discusses how T2D medications, including metformin (Met), peroxisome-proliferator-activated receptor gamma (PPAR-γ) agonists, sodium-glucose cotransporter-2 (SGLT2) inhibitors, incretins, and dipeptidyl-peptidase 4 (DPP-4) inhibitors, can improve mitochondrial function, reduce neuroinflammation and oxidative stress, and potentially inhibit ferroptosis. The connection between ferroptosis and existing treatments, including diabetes medication, are only beginning to be explored. The limited data can be attributed also to the complexity of mechanisms involved in ferroptosis and Parkinson’s disease and to the fact that the specific role of ferroptosis in Parkinson’s disease pathogenesis has not been a primary focus until recent. Despite the promising preclinical evidence, clinical findings are mixed, underscoring the need for further research to elucidate these drugs’ roles in neurodegeneration. Repurposing existing diabetes medications that have well-established safety profiles for Parkinson’s disease treatment could significantly reduce the time and cost associated with drug development and could offer a more comprehensive approach to managing Parkinson’s disease compared to treatments targeting a single mechanism.

## 1. Introduction

Two of the most frequent age-related diseases that place a significant strain on healthcare systems are Parkinson’s disease and type 2 diabetes. Parkinson’s disease affects more than 8.5 million people worldwide (in 2019, data from WHO—accessed on 11 June 2024 and estimated in 2020 to 9.4 million people according to the International Parkinson and Movement Disorder Society [1]—accessed on 2 February 2025), while T2D affects 94–96% [2] from the total of 537 million individuals with diabetes (corresponding to data from International Diabetes Federation in 2021—accessed on 11 June 2024).

The two conditions share several similarities: both are chronic in nature, both result from a reduction of a crucial substance—insulin in T2D and dopamine in PD—and both involve the destruction of a specific cell types—pancreatic β-cells in T2D and dopaminergic neurons in PD. They also share common pathological mechanisms: impaired mitochondrial function, oxidative and nitrosative stress, chronic inflammation, accumulation of misfolded proteins, ubiquitin–proteasome system dysfunction (ER stress) [3], insulin signaling impairment [4], and ferroptosis [5].

Recent studies have highlighted a bidirectional relationship between diabetes and Parkinson’s disease, wherein diabetes may increase the risk of developing Parkinson’s [6]. This connection is thought to be mediated by several factors, including impaired insulin signaling, which is crucial for both metabolic regulation and neuronal health.

The brain is a highly energy-dependent organ, primarily relying on glucose as its energy source. Most cells can reduce glucose entry in response to elevated blood glucose levels, maintaining stable internal glucose concentration. However, in diabetes, neurons lack this capability, making them susceptible to damage from hyperglycemia [7]. Insulin receptors are located in various areas of the brain, such as the cerebral cortex, hypothalamus, hippocampus, entorhinal cortex, and the substantia nigra pars compacta (SNpc) [8].

Insulin resistance (IR), a hallmark of T2D, has been implicated in exacerbating neurodegenerative processes, potentially accelerating the progression of Parkinson’s disease [9]. Insulin plays a vital role in the brain by facilitating neurotransmitter regulation [10], neuronal growth, and synaptic plasticity [11]. IR alters glucose uptake in the SNpc, affecting neuronal function by disrupting the intracellular ATP/ADP ratio and modulating potassium channels essential for dopamine release [8]. These changes influence the release of dopamine, thereby compromising dopaminergic neurotransmission, which is critical for motor control and is severely affected in PD patients.

Elevated glucose levels in IR conditions promote the production of methylglyoxal, which glycates α-synuclein (α-syn) [8]. Glycated α-syn has an increased propensity to aggregate. Hong et al. [12] further elucidated this by demonstrating that in IR conditions, there is an upregulation of α-syn expression in dopaminergic neurons. This overexpression, coupled with increased phosphorylation at Ser 129—a modification associated with α-syn aggregation—leads to the formation of protease-resistant α-syn aggregates (Lewy bodies) [13]. These aggregates disrupt normal cellular functions, including mitochondrial integrity and autophagic processes, thereby accelerating dopaminergic neuronal loss.

Mitochondrial health is essential for the survival of dopaminergic neurons, given their high energy demands. Resistance to insulin disrupts mitochondrial integrity and efficiency, further promoting neuronal death in Parkinson’s disease [14]. Sharma et al. [8] reported that IR is linked with mitochondrial dysfunction through the deregulation of key signaling pathways such as PI3K/Akt, GSK-3β, and mTOR. These disruptions lead to impaired mitochondrial biogenesis, increased production of reactive oxygen species (ROS), and compromised mitochondrial membrane potential. Insulin resistance is associated with elevated levels of ROS and oxidative stress [15]. In Parkinson’s disease, excessive oxidative stress damages dopaminergic neurons, contributing to their demise [5].

Additionally, insulin resistance enhances neuroinflammation by activating pro-inflammatory pathways [16] and over-activating microglia [17], resulting in the release of inflammatory cytokines that exacerbate neuronal damage. Sharma et al. [8] described how IR leads to the release of pro-inflammatory cytokines such as TNF-a, IL-6, and IL-1β. These cytokines can cross the blood–brain barrier (BBB) and activate microglia, the brain’s resident immune cells, promoting a chronic inflammatory state. Chronic neuroinflammation is a hallmark of Parkinson’s disease, driving the progressive loss of neurons. Activation of inflammatory pathways like NF-kB in IR conditions further promote neuronal damage and α-syn aggregation [12].

IR has an important impact on apoptotic mechanisms and autophagy within dopaminergic neurons. IR-mediated suppression of the PI3K/Akt pathway results in the activation of pro-apoptotic factors such as Bax and the inhibition of anti-apoptotic proteins like Bcl2. This shift determines neuronal apoptosis. Moreover, IR impairs autophagic processes essential for the clearance of misfolded proteins like α-syn [12].

Understanding the role of IR in dopaminergic neuronal loss highlights the potential of targeting insulin signaling pathways as a therapeutic strategy in Parkinson’s disease.

Moreover, the roles of oxidative stress, inflammation, and ferroptosis, which are common to both conditions, further underscore their interrelated nature. Chronic inflammation can exacerbate oxidative stress by increasing ROS production through the activation of immune cells and inflammatory pathways. Conversely, oxidative stress can amplify inflammation by activating signaling pathways that lead to further production of pro-inflammatory cytokines, creating a vicious cycle. The accumulation of ROS, particularly in the presence of iron, leads to lipid peroxidation, a hallmark of ferroptosis. Inflammation can also influence ferroptosis by modulating iron metabolism and ROS production. Pro-inflammatory cytokines can alter iron homeostasis, increasing the availability of free iron that catalyzes lipid peroxidation.

Unfortunately, there are no treatments available that can modify the course of Parkinson’s disease or halt its progression. The primary treatment for Parkinson’s disease involves administering levodopa (L-DOPA), a precursor for dopamine, which temporarily alleviates symptoms [18]. However, this improvement is short term, lasting only while the drug remains active in the body. Over time, the benefits of L-DOPA administration diminish as the neurons in the substantia nigra that converts it to dopamine continue to deteriorate. Additionally, long-term L-DOPA use can lead to serious side effects, including dyskinesia and dystonia [19]. Various other treatments are available to manage symptoms and improve quality of life: DOPA decarboxylase inhibitors, catechol-O-methyltransferase inhibitors, dopamine agonists, and monoamine oxidase type B inhibitors [20]. Treatment plans are typically individualized and may include a combination of approaches.

In addition to the potential benefits of the Mediterranean diet [21], reducing vitamin D deficiency [22], and avoiding substances that may trigger Parkinson’s [23], there a few options available for preventing Parkinson’s disease.

These shared pathways suggest that drugs initially developed for diabetes management might also address the underlying causes of Parkinson’s disease. Repurposing existing diabetes drugs for PD not only offers a cost-effective alternative to traditional drug development but also leverages established pharmacological and safety profiles. As research continues to uncover the molecular pathways shared by diabetes and Parkinson’s disease, the potential for diabetes medications to modify PD progression becomes increasingly plausible.

This article builds on our previous work, where we extensively discussed the involvement of ferroptosis in T2D and PD [5]. Ferroptosis is an iron-dependent form of regulated death. Cell death through ferroptosis is primarily driven by three factors: (1) elevated levels of free intracellular iron; (2) the depletion of the antioxidant defenses that manage and mitigate oxidative stress, particularly glutathione, the enzyme glutathione peroxidase (GPx4), and system xc^—^; and (3) the lipid peroxidation of membrane phospholipids that are rich in polyunsaturated fatty acids (PUFAs).

Type 2 diabetes medications, specifically GLP-1 receptor agonists and DPP-4 inhibitors, can be effectively repurposed to treat Parkinson’s disease by targeting shared pathological mechanisms such as insulin resistance, neuroinflammation, oxidative stress, and ferroptosis, thereby providing neuroprotective effects and slowing disease progression.

## 2. Antidiabetic Drugs for Parkinson’s Disease

### 2.1. Metformin

**Figure 1:**
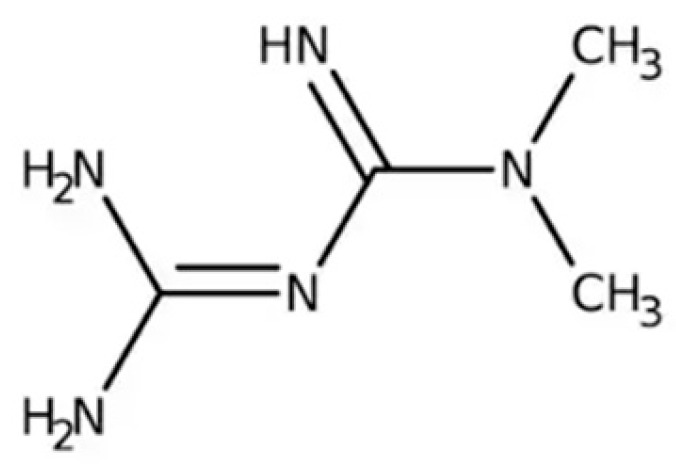
The chemical structure of metformin.

Metformin (N,N-dimethylbiguanide, Met, Figure 1) has been widely prescribed for several decades as the first-line treatment of T2D. In recent years, studies have suggested that metformin may also be effective in cancer treatment, obesity, non-alcoholic fatty liver, polycystic ovary syndrome, and metabolic syndrome and has anti-aging effects [24,25]. However, there is limited clinical evidence supporting these new applications, and scientific data are controversial. Evidence has suggested that iron dysregulation plays a role in all those pathologies, and that is why “repurposing” this mature, inexpensive, and well-known generic drug for treatment in several pathologies related with ROS production and ferroptosis might be the future.

Metformin’s primary uses and benefits in diabetes management include the acute inhibition of hepatic gluconeogenesis, promotion of glucose uptake, control of lipid metabolism, and enhancement of insulin sensitivity in peripheral tissues [26,27]. In recent years, Met has also attracted growing attention for its potential beneficial effects on various pathological conditions, including neurodegenerative diseases. Research on metformin’s impact on neuronal homeostasis has intensified, utilizing both in vitro and in vivo models, revised by Agostini et al. [20].

Met is capable of crossing the blood–brain barrier in mammals, as evidenced by its detection in the cerebrospinal fluid of rats following oral administration [28,29]. The concentration of Met was not similar in different brain regions [29].

Met is transported into hepatocytes by the organic cation transporter 1 (OCT1) [30], and, due to its positive charge, accumulates in cells and within mitochondria [31]. Here, we discuss some of the most relevant cellular pathways modulated by Met and its potential neuroprotective effects.

Met crosses the inner membrane of the mitochondria and accumulates within the organelle. It determines a mild, transient, and specific inhibition of the mitochondrial respiratory-chain complex I, without affecting the other complexes [31,32]. In Parkinson’s disease, a reduction in complex I activity has been suggested as a potential trigger for the onset of the condition [33]. As a result, cellular ATP concentrations fall, and both the ADP/ATP and AMP/ATP ratios increase, leading to energy stress, a form of metabolic stress. Initially, the cell responds by adapting to the energy stress. However, if the energy stress remains unresolved (either long-term or a severe, with very low levels of ATP), the cell might die. Scientific data on this topic are controversial: a study using a lipopolysaccharide (LPS)-induced rat model of Parkinson’s disease found that inhibition of complex I aggravated neuronal loss due to disrupted cellular homeostasis [34]. Similar results were observed in a Parkinson’s disease model when 1-methyl-4-phenyl-1,2,3,6-tetrahydropyridine (MPTP), an inhibitor of mitochondrial complex I, was administered, resulting in reduced ATP levels, which explains the drug’s detrimental effects [35]. Conversely, when rotenone, another inhibitor of the mitochondrial complex, was administered to mice, neuronal loss was diminished [36].

One adaptive response to energy stress is the activation of AMP-activated protein kinase (AMPK), a critical sensor of cellular energy status [37], through AMP binding [38]. There are six different mechanisms for AMPK activation [38]. Once activated, AMPK phosphorylates numerous proteins and enzymes to promote ATP accumulation, thereby restoring the energy balance. Several molecular mechanisms influenced by AMPK activity—such as autophagy, cell growth, and metabolism—are essential for the survival of neuronal cells and are known to be disrupted in various degenerative disorders, including Parkinson’s disease [39]. Therefore, targeting AMPK to enhance its activity is considered as a promising neuroprotective approach [40]. Met can activate AMPK through an alternative route involving the lysosomal pathway [41].

Under energy stress, AMPK activates catabolic processes and inactivates anabolic processes, primarily fatty acid synthesis and protein synthesis, in order to maintain the ATP levels [37]. AMPK inhibits, by phosphorylation, cytosolic acetyl-CoA carboxylase 1 (ACC1), the rate-limiting enzyme in fatty acid synthesis, and the outer membrane-bound mitochondrial acetyl-CoA carboxylase 2 (ACC2), which produces malonyl CoA, the allosteric inhibitor of fatty acid transport into the mitochondria for fatty acid β-oxidation [42]. In mice with alanine knock-in mutations replacing serine in both ACC1 and ACC2, which prevent their phosphorylation, liver lipid content was elevated, contributing to the development of insulin resistance and glucose intolerance [43]. Excess lipid accumulation in the liver and skeletal muscle (insulin-sensitive organs) is closely related to insulin resistance [44,45]. Mice with alanine knock-in mutations presented hyperglycemia, hyperinsulinemia, glucose, and insulin intolerance compared with wild-type controls [43]. Met improves obesity-induced insulin resistance by lowering the lipid content of the liver [43]. Lee et al. [42] showed that energy stress inhibits ferroptotic cell death partially through AMPK activation, suggesting that ACC is the effector of AMPK. As stated before, AMPK activation inhibits lipid biosynthesis, including of PUFAs-containing lipids, affecting cellular sensitivity to ferroptosis.

A higher AMP/ATP ratio directly inhibits adenylate cyclase, thereby lowering cAMP production [26]. As a consequence, PKA activity is inhibited, preventing the phosphorylation of critical proteins [46]. Additionally, cAMP levels are decreased by AMPK-dependent phosphorylation and activation of cAMP-specific 3′,5′-cyclic phosphodiesterase 4B (PDE4B) [47].

ROS production was long associated with reverse electron flux through respiratory chain complex I [48,49] and was later described in several tissues, including the brain [50]. ROS production in complex I appears to be significantly higher than in complex III [51]. Met decreases the reverse flux-related ROS production, thereby preventing oxidative stress and cell death [51].

Kukidome et al. demonstrated that Met decreases mitochondrial ROS production by induction of manganese-dependent superoxide dismutase (MnSOD) and promoting mitochondrial biogenesis [52].

Several studies have proposed that the decrease in cytoplasmic ROS production by Met (and liraglutide) results from an AMPK-mediated reduction in diacylglycerol (DAG) concentration and decreased protein kinase C (PKC) phosphorylation, leading to the inactivation of the PKC-NAD(P)H oxidase pathway in endothelial cells and thereby preventing diabetic vascular complications [53].

Bonnefont-Rousselot et al. demonstrated that Met can directly scavenge hydroxyl free radicals (though not superoxide or hydrogen peroxide) [54] and can indirectly decrease intracellular production of ROS through NADPH oxidase or the mitochondrial respiratory chain [55].

In a four-week treatment study, Pavlovic and his team found that Met enhanced the activities of Cu, Zn-SOD and catalase; increased GSH levels in erythrocytes; and reduced MDA levels in both erythrocytes and plasma of diabetic patients [56].

Met upregulates GPx4 levels by activating nuclear factor erythroid 2-related factor 2 (Nrf2) signaling pathway, decreases MDA levels, and reduces mitochondrial damage [57]. In vitro experiments have shown that Met can protect PC12 cells from cadmium-induced neurotoxicity and from H_2_O_2_-induced cell death at clinically relevant concentrations [58,59]. Additionally, Met increases the survival rate of FeCl_3_-treated PC12 cells through Nrf2 signaling pathway by reducing MDA levels [57].

Yue et al. [60] demonstrated another mechanism for metformin’s action. Their data evidenced that Met upregulated ferroportin (FPN) expression, the only cellular iron exporter, both in vivo and in vitro, by reducing its lysosomal degradation upon AMPK/FPN pathway activation. Additionally, Met reduced the expression of ferritin heavy chain, which may be responsible for the changes in ferroportin levels. As a result, hepatic iron overload was improved, malondialdehyde (MDA) levels were decreased, and intracellular levels of glutathione (GSH) and SOD were increased [60].

By contrast, Hsu et al. [61] revealed that Met can induce ferroptosis in the triple-negative breast cancer cell line (MDA-MB-231) by lowering glutathione peroxidase 4 (GPx4) levels, leading to increased accumulation of ROS.

In Parkinson’s disease, the gradual buildup of α-syn in neurons, driven by oxidative stress, specific post-translational modifications, or dysfunction in protein degradation systems (as a consequence of endoplasmic reticulum (ER) stress), leads to the formation of neurotoxic α-syn oligomers that disrupt multiple cellular pathways [62]. Consequently, metformin’s ability to enhance the autophagy pathway could potentially mitigate α-syn pathology by swiftly eliminating α-syn aggregates. Met reduced both α-syn aggregation and dopaminergic neuron loss in a *C. elegans* Parkinson’s disease model exposed to 6-hydroxydopamine [63]. Met can reduce the levels of phosphorylated α-syn at serine 129 by activating protein phosphatase 2A (PP2A) via AMPK-dependent and independent pathways [64]. The formation and accumulation of phospho-Ser129 α-syn in Lewy bodies are likely to play a significant role in the pathogenic processes of Parkinson’s disease [65]. Administering Met to mice previously injected with MPTP resulted in a reduction of pathological α-syn levels, activation of autophagy, restoration of dopamine levels, and improvement in motor performance [66].

Interestingly, methylglyoxal (MGO) can covalently bind to α-syn, similar to other proteins, triggering its oligomerization in various in vitro and in vivo models and thereby worsening neurodegeneration [67]. MGO modifies proteins through non-enzymatic glycation, leading to the formation of advanced glycation end-products (AGEs). Metformin’s use in treating T2D is attributed to its ability to act as a scavenger of MGO. Agostini et al. speculated that Met might also prevent the accumulation of neurotoxic α-syn aggregates through a similar mechanism [20]. They proposed that Met could also react with other aldehydes generated from oxidative stress and lipid peroxidation, such as 4-hydroxynonenal (4-HNE), MDA, or aldehydes accumulating from altered monoamine catabolic pathways [20]. This speculation is supported by a recent study in a rotenone-induced Parkinson’s disease mouse model, where the co-administration of Met reduced nigral levels of 4-HNE and MDA (thus, lipid peroxidation) and decreased α-syn accumulation and dopaminergic neuron degeneration in the substantia nigra pars compacta (SNpc) [68]. Met may also reduce plasma levels of MGO by enhancing its detoxification through the upregulation of glyoxalase activity, which is part of its degradation pathway. This effect is likely due to an increase in glutathione production [69].

Recent studies involving MPTP-induced Parkinson’s disease in mice have demonstrated that Met enhances muscular activity and locomotion. Its neuroprotective effects are attributed to the inhibition of α-syn phosphorylation and aggregation, the induction of neurotrophic factors [66], the reduction of oxidative stress, and the protection of the dopaminergic neurons [70]. Clinical trials conducted on the Taiwanese population showed that long-term treatment with Met was more effective that acute treatment and lowered the risk of developing PD alongside T2D [70].

The first study to report the association between metformin’s cytoprotective effects and β-cell ferroptosis in vivo was conducted in 2023, laying ground for future research. Sun et al. [71] demonstrated in two diabetic models (low-dose streptozotocin and high-fat diet-induced diabetes, as well as db/db mice) that Met decreased the lipid-related ROS overproduction, which was less obvious when RSL3 (a ferroptosis activator) was injected. Met enhanced the pancreatic GSH/GSSG ratio and the level of GPx4, while reducing the expression level of acyl-CoA synthase long-chain family member 4 (ACSL4, a biomarker and contributor to ferroptosis [72]), in mouse islets from the two diabetes models. Injection with RSL3 significantly diminished Met effects, indicating that its antiferroptotic effects are related to the modulation of the GPx4/ACSL4 axis.

Conflicting results have been published regarding the use of Met in neurodegenerative diseases, and these results need to be interpreted with caution because of the frequent use of Met worldwide. Ping et al. concluded in a meta-analysis that Met “plays a neutral effect on the risk of neurodegenerative diseases in general” [73]. However, a subgroup analysis comprising three cohort studies showed an increase of PD risk by 66% [73].

In a 6-hydroxydopamine-induced Parkinson’s disease animal model, Ryu et al. found that Met did not affect dopaminergic cell death but regulated changes in astrocyte-specific genes that lead to changes in microglial and astrocyte activation [74].

In Table 1, we summarize the primary mechanisms by which metformin impact Parkinson’s disease (all the references are included in the above text).

Further research is needed to clarify Met’s role in neuroprotection and its potential repurposing for conditions associated with oxidative stress and ferroptosis. As the scientific community continues to explore these avenues, Met’s potential as a multi-faceted therapeutic agent remains an exciting prospect.

### 2.2. Incretins and Incretin Mimetics

There is increasing evidence that the homeostasis of the gut–brain axis is involved in progression of neurodegenerative diseases, such as Alzheimer and Parkinson’s disease. Insulin-mediated glucose control is critical for the brain because glucose is its most important source of energy [75]. Thus, insulin dysregulation might be a shared pathological process that associates patients with T2D with a higher incidence of PD [76].

The gut–brain axis is defined as the bidirectional communication between the brain and the gut [77]. This connection includes neuronal, immune, and endocrine pathways and involves several cytokines, hormones, neurotransmitters, and neuromodulators. The enteric nervous system, in this way, connects the intestine with the central nervous system.

According to Baggio and Drucker [78], incretins are “hormones that are secreted from the gastrointestinal tract into the circulation in response to nutrient ingestion that enhance glucose-stimulated insulin secretion”. Incretins also slow gastric emptying after eating, promote pancreatic beta cell proliferation, and reduce food intake [79].

Two of the most known incretins are glucagon-like peptide -1 (GLP-1) and gastric inhibitory polypeptide (GIP), the latter later renamed glucose-dependent insulinotropic polypeptide.

GLP-1 is a small insulinotropic peptide, mainly secreted by the intestinal L cells located mainly in the distal ileum and colon, that plays an important role in glucose regulation in the gut–brain axis. It is a post-translational proteolytic product of the proglucagon gene and is quickly inactivated and degraded (with a plasma half-life is 1–2 min) by the endopeptidase DPP-4. As a result, the concentration of intestinally produced GLP-1 is very low, insufficient to activate central GLP-1receptors (GLP-1Rs) [80]. Therefore, GLP-1 must also be secreted in the central nervous system. Indeed, one source for GLP-1 is the nucleus tractus solitarius neurons of the brainstem [81], the cerebral cortex and hippocampus [82], the microglia [83] and astrocytes [84] and directly in the hypothalamus (a minor amount) [78].

GLP-1R is a class B family G-protein-coupled receptor having a seven-transmembrane-domain, the same class as glucagon and GIP receptors [78]. GLP-1Rs are present in pancreas, lung, intestine, stomach, kidney, heart, and in several regions of the brain [85]. Pancreatic, cardiac, and brain receptors have the same amino acid sequence and ligand specificity [86]. In the brain, GLP-1Rs are extensively present, suggesting that GLP-1 could play a role in managing various neurological and cognitive functions beyond glucose metabolism regulation [82].

In the pancreas, binding of GLP-1 to its receptor leads to adenylate cyclase activation and cAMP production. Subsequently, insulin secretion is stimulated by several mechanisms: activation of cAMP/PKA-dependent and independent-signaling pathways, increases in cellular Ca^2+^ levels, β-cell membrane depolarization, and increases in mitochondrial ATP synthesis. This leads to further membrane depolarization and exocytosis of insulin storage granules due to increases ATP and intracellular Ca^2+^ levels [40]. GLP-1 also promotes insulin gene transcription, mRNA stability, and biosynthesis [78]; confers glucose sensitivity to glucose-resistant β-cells [87]; inhibits glucagon; and stimulates somatostatin secretion [78].

In the brain, GLP-1 exerts anti-inflammatory and anti-apoptotic effects, thereby preventing neuronal damage [88]. GLP-1R is not highly expressed in glial cells, but their density increases following an inflammatory response in brain, suggesting their involvement in regulating inflammation. GLP-1 is even regarded as an anti-inflammatory cytokine that reduces the release of pro-inflammatory cytokines [89].

**Figure 2:**
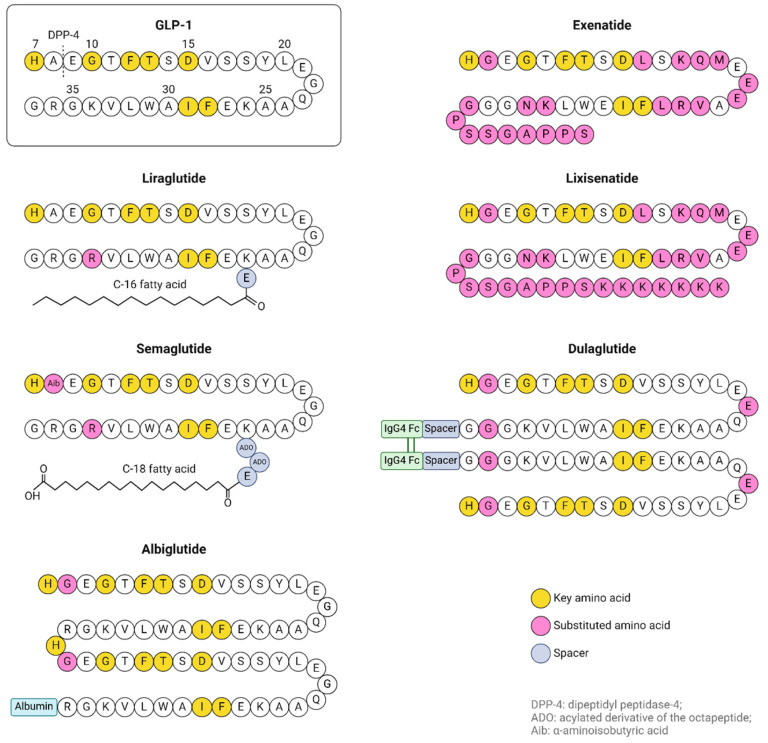
The molecular structures of GLP-1 and some of the FDA approved GLP-1 receptor agonists. Created in https://BioRender.com.

GLP-1R agonists (also known as GLP-1 analogues or incretin mimetics) have been approved by the US Food and Drug Administration for the treatment of T2D not only for their effect on glucose regulation but also for their beneficial effect on beta cell function [85] (Table 2 and Figure 2).

GLP-1R agonists stimulate β-cell proliferation and neogenesis and inhibit β-cell apoptosis [78]. These agonists do not cause insulin desensitization with prolonged use, as they do not activate insulin receptors in individuals with normal blood glucose levels, thereby preventing hypoglycemia [90]. They are resistant to DPP-4 degradation (Exenatide has a half-life of 2.4 h, liraglutide—13 h, semaglutide—7 days [84]), and some can pass the BBB [91]. Exenatide and lixisenatide were demonstrated to cross the BBB [92], while liraglutide and semaglutide access the brain via small circumventricular organs [90]. It has been proposed that intranasal administration of GLP-1R agonists and DPP-4 inhibitors (gliptins) or novel nano-formulations could enhance their ability to cross the BBB via the olfactory region, targeting the cerebrospinal fluid [93].

The effects of GLP-1R agonists on the brain occur independently of their role in controlling blood sugar levels [94]. Liraglutide and lixisenatide promote adult neurogenesis [91,95], and exendin-4 increases the rates of neuronal progenitor cells in induced Parkinson’s disease animal models [96].

GIP is produced and released by K-cells in the intestine, predominantly found in the duodenum and proximal jejunum. GIP secretion is triggered by nutrient intake (fat is the strongest stimulus for GIP release in humans, while in rodents, carbohydrates are) and is driven by the rate of nutrient absorption rather than just the presence of nutrients in the gut [78].

GIP has a half-life of less than 2 min in rodents [97] and 5–7 min in healthy subjects and T2D patients [98] because it is also a target for DPP-4.

The GIP receptor (GIPR) is also a member of the seven-transmembrane-spanning, G-protein-coupled receptor subfamily and is present in almost every tissue, including several regions of the CNS [78].

Upon binding of GIP to its receptor, cAMP and intracellular Ca^2+^ are increased, and PI-3K, PKA, PKB, MAPK, and phospholipase 2 pathways are activated.

In pancreatic β-cells, GIP has similar actions as GLP-1: it enhances insulin secretion, regulates insulin gene transcription and biosynthesis, stimulates cell proliferation, and improves β-cell survival [78]. Additionally, GIP reduces ER stress in islet cells in vitro [99]. ER stress results from the depletion of calcium stores in the ER, leading to the malfunction of local chaperones and the accumulation of unfolded or misfolded proteins within the ER lumen.

In the CNS, GIP is expressed in the hippocampus, and GIPR is expressed in the cerebral cortex, hippocampus, and olfactory bulb [78].

While direct evidence linking GLP-1 receptor agonists and GIP to ferroptosis inhibition is limited, their known effects on reducing oxidative stress, improving mitochondrial function, and exerting anti-inflammatory actions suggest potential indirect influences on ferroptotic pathways.

#### 2.2.1. Preclinical Evidence for GIP and GLP-1R Agonists Usage in Parkinson’s Disease Treatment

GLP-1 receptor analogues exhibit several pro-cognitive effects, as reviewed by Reich and Holscher [100]: they protect the synapses and promote synaptogenesis, enhance hippocampal synaptic plasticity, rescue cognitive decline (including learning and memory consolidation), prevent Ca2+ overload in neurons, protect nigrostriatal neurons, replenish dopamine production, suppress ER stress, exert anti-inflammatory effects, protect against external oxidative stress and ROS production, and mitigate mitochondrial dysfunction [100].

GLP-1 and Exenatide have been shown to protect human neuroblastoma cells (SH-SY5Y) from H_2_O_2_ induced cell death in a dose-dependent manner [101,102]. The administration of the long-acting GLP-1R agonist exendin-4 (Ex-4) reduced brain damage, protected dopaminergic neurons against degeneration, and improved motor function in a MPTP mouse model of Parkinson’s disease [101].

Li et al. [103] showed that GLP-1R agonists protect neurons under oxidative stress and reduce ischemia-induced damage. Studies using Ex-4 in cerebral ischemia model rats demonstrated improved oxidative parameters and neuronal protection [104].

Shen et al. [105] demonstrated that Ex-4 administration in streptozotocin-induced diabetic mice had beneficial effects on kidney injury. Ex-4 significantly reduced iron overload, oxidative stress, and ACSL4-driven lipid peroxidation in diabetic kidney tubules, while also mitigating the reduction in GPx4 expression and GSH content.

In another study, liraglutide was shown to reduce infarct volume and oxidative stress parameters by stimulating GLP-1Rs in stroke rat models [106]. Pretreatment with liraglutide significantly lowered MDA levels, a marker of lipid peroxidation, while enhancing GSH concentrations and SOD activity in the brain [107].

An et al. [108] demonstrated that liraglutide administration reduced MDA levels and significantly increased SOD and GPx levels in serum of db/db mice. Similar results were observed in the hippocampus, especially in the CA3 (cornu Ammonis) region. Iron overload in the brain contributes to ROS and lipid peroxide generation and is associated with cognitive impairment in diabetes and Parkinson’s disease. In db/db mice, iron content was markedly elevated in the hippocampus, especially in the CA1, CA3, and DG (dentate gyrus) regions, while H-ferritin (iron storage protein) was decreased, transferrin receptor (TfR1) was elevated, and the FPN1 (the only iron exporter) was decreased [108]. An et al.’s studies showed that liraglutide treatment decreased iron content and improved iron metabolism in db/db mice. While the mitochondria aspect was consistent with ferroptosis (reduced and shrunken mitochondria), ACSL4 was increased, and GPx4 and cystine transporter system (SCL7A11) were decreased in db/db mice and more pronounced in the CA3 region of the hippocampus. Liraglutide administration reversed these observations, according to An et al. [108].

**Figure 3:**
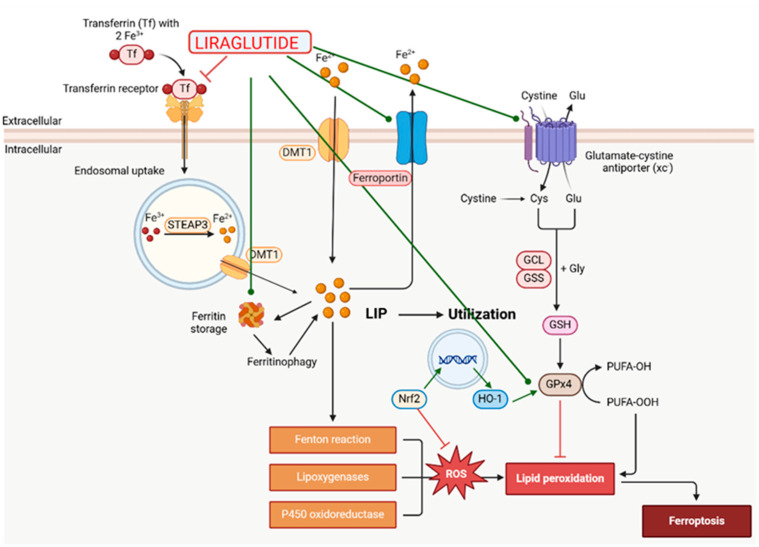
Liraglutide downregulates TfR1 expression, upregulates the expression of FPN1 and SLC7A11 (part of the glutamate–cysteine antiporter) elevating GSH concentrations, activates the Nrf2/HO-1 pathway and elevates the expression of GPx4. Adapted from [4]. Created in https://BioRender.com.

Song et al. [109] demonstrated that in db/db mice, hepatocytes treated with liraglutide inhibited ROS production by upregulating SOD and GPx4 activity and improving GSH levels. Liraglutide also reduced MDA and 4-HNE levels, as well as the expression of NADPH oxidase 4 (NOX4) in the hepatocytes of db/db mice. These results indicate that oxidative stress is improved by upregulating antioxidant capacity and decreasing lipid peroxidation. Treatment with liraglutide reduced the iron content of the liver by downregulating TfR1 expression and upregulating FPN1 expression, which are iron-related transport proteins. Liraglutide also enhanced the expression of GPx4 and SLC7A11 and increased the expression of antioxidant signaling factors Nrf2 and heme oxygenase 1 (HO-1), suggesting that the drug attenuates ferroptosis in the liver of db/db mice (Figure 3).

Previous studies have shown that liraglutide activates the Nrf2/HO-1 signaling pathway in neuronal cells in the brains of diabetic mice [110], making this signaling pathway the most important mechanism to counteract ferroptosis in both tissues upon liraglutide treatment.

Duarte et al. demonstrated that liraglutide reduced central inflammation and oxidative stress in Alzheimer’s disease mice, possibly via the stimulation of glucose-6-phospate dehydrogenase (G6PDH) and the regulation of NADPH and GSH concentrations [111]. Liraglutide also recovered mitochondrial membrane integrity and complex I activity, improving mitochondrial function and lowering superoxide anion formation, probably via NF-kB inhibition [111].

Liraglutide activates the PI3K/AKT and ERK pathways, which can regulate the expression of Bcl-2 family. Bcl2 and Bcl-xL proteins are important antioxidant proteins that scavenge free radicals and inhibit the formation of superoxide anion [112].

Spielman et al. [113] demonstrated that GLP-1 and GIP could also have an indirectly neuroprotective effect through the regulation of glial cell functions. The researchers also demonstrated the presence of GLP-1R and GIPR in human microglia and astrocytes for the first time. Microglia are responsible for protecting the neurons by eliminating toxins and pathogenic molecules, as well as providing nutrients and trophic factors to the neurons. Microglia are chronically activated in Parkinson’s disease [114] and, as a result, the number of available microglia is reduced as they are destroyed by apoptosis [115]. GLP-1, in particular, reduces intracellular ROS, decreases nitric oxide (NO) production, and upregulates the expression of GPx-1 and SOD in murine BV-2 microglia. However, there was a limitation in the study because the activated microglia expressed DPP-4 enzyme, which reduced the concentration of both GLP-1 and GIP. Consequently, the modest observations may be attributed to partially activated microglia or resting microglia. Instead, other effects of the treatment on microglia are more significant, such as reducing apoptosis and inducing trophic factors.

Preclinical studies involving GIP and GLP-1R agonists have shown promising results in preclinical models of Parkinson’s disease, indicating their potential as a therapeutic target for both symptomatic relief and disease modification.

#### 2.2.2. Clinical Evidence for GIP and GLP-1R Agonists Usage in Parkinson’s Disease Treatment

Following encouraging results from animal models and experimental data, additional research in humans was required to assess whether current diabetes treatment could alter the progression of Parkinson’s disease in patients.

In 2013, Aviles-Olmos et al. [116] published the results of a single-blind trial using Exenatide for 12 months in 45 patients with moderate Parkinson’s disease. The results showed both cognitive and motor benefits in Exenatide-treated patients, and these benefits were still observed after an additional 12 months [117]. However, patients experienced weight loss accompanied by worsening dyskinetic symptoms at 12 and 14 months.

In a single-center, randomized, double-blind, placebo-controlled trial, Athauda et al. administered Exenatide to 62 patients with moderate Parkinson’s disease over a period of 48 weeks [118]. The results indicated that patients experienced improvement in the Movement Disorder Society-Unified Parkinson’s Disease Rating Scale Part III (MDS-UPDRS-3). Moreover, the treatment benefits persisted beyond the washout period and even exceeded those at the 48-week checkout. In 2019, the same group published another analysis showing that the best results of the trial were obtained in patients with lower MDS-UPDRS2-2 scores and the tremor-dominant phenotype, and in patients with less than 4 years duration of disease [119].

In conclusion, until 2020, the clinical trials showed low-certainty evidence, suggesting improvements in motor impairment and little or no effect on health-related quality of life upon administration of Exenatide for people with Parkinson’s disease for a minimum 11 months. The fact that the improvements persisted for several weeks after patients stopped taking the drug suggests that “the drug modified the disease process in some way” [119]. Larger sample sizes, a longer period of follow-up of participants, consideration of the progressive nature of Parkinson’s disease, and multi-center, placebo-controlled studies are needed, as concluded by Mulvaney et al. [120].

A phase 2, randomized, double-blinded, placebo-controlled trial of liraglutide treatment in PD showed that clinical features of Parkinson’s disease, including mobility, non-motor symptoms, and quality of life, were improved [121].

Several other clinical trials have been conducted on Exenatide (a phase 3 trial [122]) and on other incretins’ (liraglutide, semaglutide, or lixisenatide) effects in Parkinson’s disease [84].

Currently, new GLP-1/GIP dual receptor agonists are being developed that are more effective for diabetic patients than using GLP-1 or GIP alone. These dual agonists fully utilize the pharmacological benefits of both receptors while minimizing side effects [123] and have shown better results in mouse models of Parkinson’s disease [124,125]. The dual agonists have an enhanced capability to penetrate the BBB, which is crucial for drugs aimed at reducing neurodegenerative disorders in the central nervous system [126]. However, similar to individual GLP-1 or GIP receptor agonists, there are limited data regarding their role in reducing oxidative stress or on influencing ferroptosis. Tirzepatide (Tirze) was approved by the FDA for treatment of T2D and has shown neuroprotective effects in diabetic rats [127]. It reduces the MDA content, markedly elevates the GSH content, and significantly reduces the ATF-4 and CHOP levels (ER stress associated markers) in the brain of colistin-treated rats (colistin-treatment is associated with serious neurotoxicity and nephrotoxicity, which restricts the use of the antibiotic) [128].

The AP5 dual receptor agonist significantly decreased MDA levels, increased SOD activity, and inhibited ROS production in the myocardial tissue of diabetic mice [129].

The DA-JC1 dual receptor also showed neuroprotective properties against H_2_O_2_-induced stress in SH-SY5Y cells that expressed GLP-1 and GIP receptors [130]. It prevented ROS production and DNA damage induced by the oxidative stress.

While no GPL-1/GIP/Glucagon receptor agonists are currently approved for public use, several have progressed to clinical trials for the treatment of metabolic disorders [131]. A peptide triple receptor agonist incorporating GLP-1, GIP, and glucagon actions, termed Triagonist, was tested in neuronal SH-SY5Y cultures and demonstrated neurotrophic and neuroprotective actions against H_2_O_2_-induced oxidative stress [132].

In conclusion, incretin mimetics like Exenatide are at the forefront of being repurposed for PD due to their stronger evidence base in neuroprotection and BBB permeability. In Table 3, we summarize the primary mechanisms by which incretin mimetics impact Parkinson’s disease (all the references are included in the above text).

### 2.3. DPP-4 Inhibitors (Gliptins)

DPP-4 inhibitors increase GLP-1 and GIP levels, prolonging their half-life, and are used in the treatment of T2D [133,134]. They exhibit very low concentrations in the brain, likely because they do not cross the BBB, which may limit the application of most of them [135].

**Figure 4:**
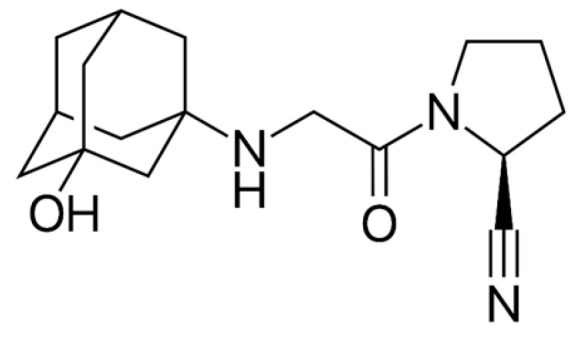
The chemical structure of vildagliptin. Reproduced from Wikimedia Commons, https://commons.wikimedia.org/wiki/File:Vildagliptin.svg. Accessed 12 April 2024.

**Figure 5:**
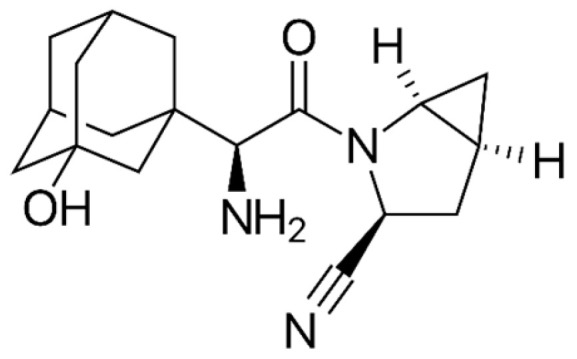
The chemical structure of saxagliptin. Reproduced from Wikimedia Commons, https://commons.wikimedia.org/wiki/File:Saxagliptin_structure.svg. Accessed 12 April 2024.

**Figure 6:**
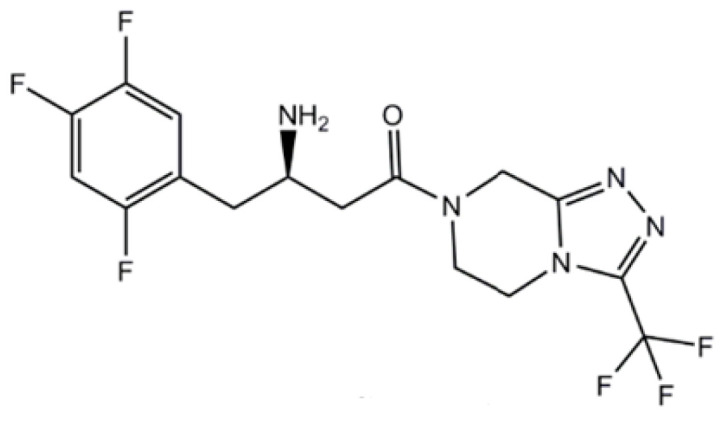
Chemical structure of sitagliptin. Reproduced from [149].

In Table 4, we present the marketed DPP-4 inhibitors approved for use as monotherapy. In Figure 4, Figure 5 and Figure 6, we present the chemical structures of three of the DPP-4 inhibitors used in experiments regarding Parkinson’s disease. Additionally, some are available on the market in combination therapies.

Most studies investigating the use of DPP-4 inhibitors in Parkinson’s disease treatment have shown that they suppress inflammation and apoptosis. However, the generation of ROS and reactive nitrogen species (RNS) is associated with oxidative stress and lipid peroxidation, suggesting that ferroptosis may also be involved, given the high concentrations of iron in the brain.

Advanced glycation end-products (AGEs), formed because of high glucose concentrations, are involved in inflammation and cell migration processes in several diseases. AGEs serve as ligands for the receptors for advanced glycation end-products (RAGE), which are widely distributed in cells, including neurons, and whose expression is upregulated in response to inflammation [136]. Nuclear factor kB (NFkB) is the primary signal transduction molecule activated by AGEs, initiating the expression of other inflammatory molecules. The RAGE-NFkB signaling pathway also regulates inducible nitric oxide synthase (iNOS), responsible for synthesizing nitric oxide (NO) and generating other ROS and RNS [136]. Additionally, ROS production is induced by the activation of NADPH oxidase, following NFkB transcriptional activation by neutrophils activated and infiltrating the brain from the blood, as well as through dopamine catabolism [136].

Vildagliptin is more effective at increasing GLP-1 concentration compared to other DPP-4 inhibitors [137]. In addition to its role in preserving β-cell function by suppressing oxidation, ER stress, apoptosis, and inflammation [138,139], it improves memory and cognition by modulating brain inflammation, mitochondrial disfunction, and oxidative stress [136,140,141]. In db/db mice, vildagliptin suppressed ER-stress by downregulating the C/EBP homologous protein (CHOP), tribbles homolog 3 (TRIB3) and activating transcription factor 4 (ATF-4) [142]. ER stress can lead to the production of ROS in the ER and mitochondria, with both ROS production and protein misfolding prompting the cell to undergo apoptosis [143]. Activated CHOP contributes to ROS production, resulting in oxidative damage and disruption of iron balance. Treatment with CHOP siRNA (small interfering RNA) significantly reduces ROS production and alterations in iron regulatory proteins [144].

In a rotenone-induced Parkinson’s disease experimental model [136], vildagliptin reduced oxidative/nitroactive stress in the striatum and prevented the reduction of dopamine content in the striatum. The inhibitor blocked the RAGE/NFkB cascade, resulting in lowered concentrations of Nrf2 and TBARS (thiobarbituric acid-reactive substances), a marker for lipid peroxidation. Similar results were obtained by Matsui et al. [145]. The study demonstrated that vildagliptin decreased iNOS transcription, thereby reducing peroxynitrite formation, and lowered neutrophilic myeloperoxidase (MPO) activity, which could convert nitrite into free radical nitric oxide, reducing oxidative and nitrosative stress.

Saxagliptin also showed a neuroprotective potential in an animal model of rotenone-induced Parkinson’s disease, preventing dopaminergic neurons loss in the SNpc and striatum. The study evidenced that in rotenone-exposed rats, DPP-4 was elevated, suggesting that the depletion of GLP-1 in the brain could lead to loss of dopaminergic neurons, and the decline of dopamine and that GLP-1 was a survival factor for dopaminergic neurons [146]. ROS produced by resident-activated microglia and recruited neutrophils, along with dopamine oxidation products and peroxynitrite, formed from the reaction of superoxide anion and NO (facilitated by iNOS), contributed to increased lipid peroxidation. In the same study, saxagliptin administration reduced lipid peroxidation in the striatum by decreasing NrF-2 levels. Nrf-2 levels were raised due to rotenone exposure (and also in normal animals), indicating its antioxidant activity. Saxagliptin reduced free radicals production by suppressing intracellular adhesion molecule-1 (ICAM-1), an adhesion molecule that mediated neutrophil infiltration, and prevented the formation of RNS through both direct and indirect iNOS inhibition [146]. Rotenone treatment diminished mitochondrial complex I activity, leading to a decline in ATP synthesis and reducing Bcl-2 levels. Along with the production of ROS and RNS, these were decisive steps in the permeabilization of the outer mitochondrial membrane and the release of cytochrome c. Saxagliptin reversed all these effects [146].

Sitagliptin treatment of rotenone-induced Parkinson’s disease albino rats showed positive effects in preventing PD progression because of its “antiapoptotic, neurogenic, neurotrophic and anti-inflammatory activities” [147] but increased tau phosphorylation in T2D rats and primary neuron cultures [148]. Further studies need to be performed to clarify the precise mechanism connecting sitagliptin use to the risk of PD.

In a retrospective cohort study in Taiwan, saxagliptin, sitagliptin, linagliptin, and vildagliptin did not show an increased risk of PD in T2D patients. In animal models, all these DPP-4 inhibitors have shown positive effects, but the doses used were much higher than those used in humans [134]. The inability of most DPP-4 to cross BBB and their harmful secondary effects may limit their use. Only teneligliptin, trelagliptin [150], and omarigliptin [134] seem to cross the BBB, but further studies are needed.

Data from case-control studies [151], longitudinal cohort studies [152], and retrospective studies [153] have shown a decreased incidence of PD in patients receiving DPP-4, both diabetic and nondiabetic.

Although direct evidence linking DPP-4 inhibitors to ferroptosis inhibition is limited, their known effects on reducing oxidative stress and inflammation suggest potential indirect influences on ferroptotic pathways. The potential for DPP-4 inhibitors to be used in combination with other treatments for Parkinson’s disease is an area of interest. Their ability to enhance GLP-1 activity could complement other therapeutic strategies.

Table 5 presents a summary of the key mechanisms by which DPP-4 inhibitors influence Parkinson’s disease, with all the relevant references provided in the preceding text.

Both incretin mimetics and DPP-4 inhibitors enhance GLP-1 activity. We chose to discuss them separately as their chemical structure is different. For a better understanding, we will summarize their similarities and differences, particularly in terms of neuroprotective efficacy, BBB permeability, and impact on ferroptosis-related pathways (Table 6).

In conclusion, incretin mimetics (GLP-1 receptor agonists) generally demonstrate greater neuroprotective efficacy, better permeability across the blood–brain barrier, and a more consistent impact on ferroptosis-related pathways compared to DPP-4 inhibitors.

Incretin mimetics have shown more promising results in both preclinical and clinical studies, making them more favorable candidates for further investigation and potential therapeutic use in Parkinson’s disease. Conversely, DPP-4 inhibitors, may offer modest benefits, but their limited ability to penetrate the BBB and less consistent effects on ferroptosis-related pathways temper their utility in this context.

### 2.4. Peroxisome-Proliferator-Activated Receptor Gamma (PPAR-γ) Agonists

Thiazolidinediones (TZDs) are a significant class of second-line agents used to lower blood glucose [154] by increasing insulin sensitivity in patients with type 2 diabetes [76,155]. They primarily function by binding to the nuclear peroxisome-proliferator-activated receptor gamma (PPAR-γ) in the muscle, liver, and adipose tissue, thereby increasing glucose uptake in these areas and decreasing glucose production in the liver [156]. PPAR-γ is also expressed in many regions of the brain, including the dopaminergic cells in basal ganglia [157].

Members of the class marketed today are pioglitazone (Actos), rosiglitazone (Avandia), and lobeglitazone (Duvie, approved only in Korea) (Wikipedia, accessed 11 March 2024).

The reasoning behind investigating TZDs for Parkinson’s disease originates from their mechanism of action, which involves the activation of PPAR-γ. PPAR-γ plays a role in regulating inflammation and mitochondrial function, both of which are relevant to the pathophysiology of Parkinson’s disease. Activation of PPAR-γ can lead to the upregulation of antioxidant genes, potentially mitigating lipid peroxidation.

Currently, there is limited direct evidence linking TZDs to the modulation of ferroptosis specifically. However, given the interconnected nature of oxidative stress and inflammation, it is plausible that TZDs could indirectly affect iron homeostasis, thereby impacting ferroptotic processes.

**Figure 7:**
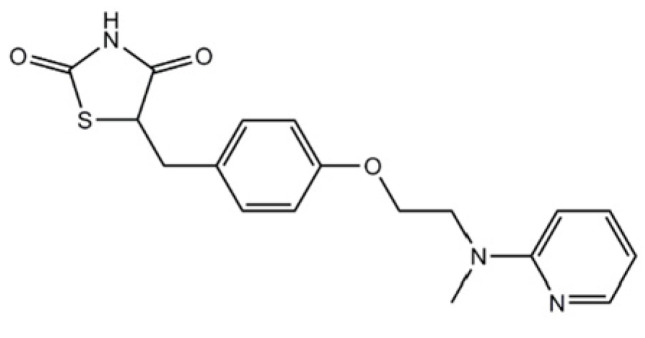
The chemical structure of rosiglitazone. Reproduced from [149].

Rosiglitazone (RSG; its chemical structure is presented in Figure 7) has been shown to reduce mitochondrial damage in tubular epithelial cells after ischemia/reperfusion injury in mouse kidneys [158]. Treatment with RSG led to an increase in GSH levels and a decrease in MDA and 4-HNE levels, indicators of lipid peroxidation. Additionally, markers of ferroptosis were significantly modified: GPx4 levels increased, while ACSL4 levels significantly decreased [158].

Lai et al. [159] demonstrated that RSG treatment counteracted hypoxia-induced ferroptosis in trophoblasts by modulating Nrf2 (a transcription factor that regulates intracellular stress; GPX4 is a downstream target gene for Nrf2). RSG also reduced the effect of erastin in HTR-8/SVneo cells.

Several studies have demonstrated that TZDs are inhibitors of ACSL4 through PPAR-γ activation. In reflux esophagitis in rats, treatment with RSG significantly reduced iron accumulation and lipid peroxidation [160]. RSG has also proven efficient in inhibiting ferroptosis in acute kidney injury in mice by downregulating ACSL4 levels [161]. In cases of lung ischemia–reperfusion injury, RSG reduced lipid peroxidation while increasing GSH and GPx4 levels [162]. Li et al. [163] showed that RSG pretreatment in mice abolished ferroptosis and protected against ischemia-reperfusion injury in intestine.

In Alzheimer’s disease (AD), TZDs significantly inhibit ACSL4 by directly preventing the incorporation of polyunsaturated fatty acids (PUFAs) into cellular lipids in a PPAR-γ-independent manner, thus inhibiting ferroptosis. RSG had the strongest inhibitory effect compared to pioglitazone (PIO) or troglitazone [164].

**Figure 8:**
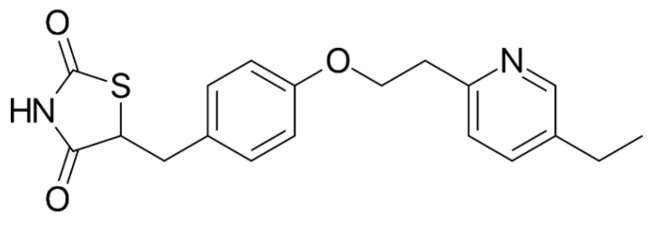
The chemical structure of pioglitazone. Reproduced from Wikimedia Commons, https://commons.wikimedia.org/wiki/File:Pioglitazone.svg. Accessed 12 April 2024.

Breidert et al. [165] and Dehmer et al. [166] demonstrated that PIO has a protective role against neurodegeneration in an MPTP mouse model of Parkinson’s disease. Pioglitazone (PIO; its chemical structure is presented in Figure 8) acts via PPAR-γ activation, reducing iNOS induction and NO-mediated toxicity [166]. In a lipopolysaccharide model of Parkinson’s disease (a model for inflammation-induced dopaminergic neurodegeneration), PIO reduced inflammation, mitochondrial dysfunction, and oxidative stress while enhancing dopamine concentration, thereby improving nigral dopaminergic neuronal loss and reducing microglial activation [167]. PIO treatment also reduced oxidative stress caused by MPTP treatment in rats, evidenced by decreased MDA levels and increased GSH levels [168].

In a human normal hepatocyte cell line (QZG), RSG inhibited ROS production by inhibiting the activation of PKC triggered by high glucose levels. It also increased the expression of Nrf2 (a key antioxidant transcription factor) and HO-1 (an antioxidant enzyme), thereby lowering ROS concentrations [169].

RSG showed protective effects in human neuroblastoma SH-SY5Y cells in an acetaldehyde-induced severe Parkinson’s disease-like syndrome [170]. The protective effects were linked to its ability to stimulate the expression of antioxidant enzymes, such as SOD and catalase, among other effects [170].

PPAR-γ agonists provide comprehensive neuroprotection by reducing oxidative stress, inhibiting lipid peroxidation, modulating inflammatory responses, and enhancing mitochondrial function. However, clinical studies have produced inconsistent results, likely due to differences in methodology, study population, and follow-up durations. As a result, clinical trials evaluating TZDs as a treatment for PD have been discontinued. Recently, concerns have been raised about possible adverse cardiovascular effects and an increased risk of bladder cancer associated with TZD use (159,160).

### 2.5. Sodium–Glucose Cotransporter-2 Inhibitors (SGLT2i)

**Figure 9:**
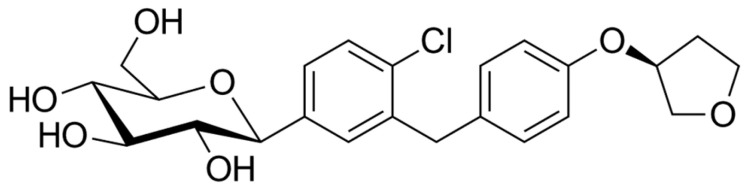
The chemical structure of empagliflozin. Reproduced from Wikimedia Commons, https://commons.wikimedia.org/wiki/File:Empagliflozin.svg. Accessed 12 April 2024.

**Figure 10:**
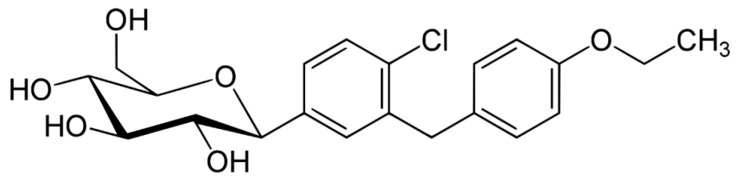
The chemical structure of dapaglifozin. Reproduced from Wikimedia Commons, https://commons.wikimedia.org/wiki/File:Dapagliflozin_structure.svg. Accessed 12 April 2024.

Some studies have indicated that SGLT2i may help reduce oxidative stress and inflammation. It has been suggested that they act as indirect antioxidants by lowering free radical production [171], boosting antioxidant systems such as glutathione and SOD [172,173,174], suppressing pro-oxidants like thiobarbituric acid-reactive substances, reducing nicotinamide adenine dinucleotide phosphate oxidase 4 (NOX4) [175], and lowering glucose-induced oxidative stress [176]. Asil et al. [177] demonstrated that treatment with SGLT2i, specifically empagliflozin and dapagliflozin (their chemical structures are presented in Figure 9 and Figure 10) in diabetic patients, decreased NO production and nitrosative stress and reduced ferroptosis by elevating GPx4 and suppressing ACSL4 levels. Dapagliflozin also decreased the production of superoxide [178]. Sharma et al. recently showed that SGLT2i decreased blood MDA and NO levels and increased GSH levels in T2D [179].

However, direct evidence of these effects specifically in the context of Parkinson’s disease is limited. Current research offers varying perspectives on how SGLT2i influences the risk of PD, as this subject is relatively new and still in the early stages of exploration. Wu et al. [180] were the only researchers to report an association between SGLT2i use and a reduced risk of PD. A recent population-based cohort study form Korea found that the use of SGLT2i notably decreased the risk of PD in individuals with T2D [181]. In contrast, Liu et al. evidenced that SGLT2i users had an increased risk of PD [182].

These conflicting results highlight the need for further investigation to understand the underlying mechanisms, potential confounding factors, and the role of individual patient characteristics to provide a more definitive conclusion.

### 2.6. Meglitinides (Glinides)

Meglitinides are used to manage T2D by stimulating the proinsulin secretion, and they have not been extensively studied in the context of PD risk. As their primary effect is on glucose metabolism, they have little impact on pathways typically associated with neurodegeneration, such as inflammation or oxidative stress.

Approved glinides for diabetes treatment include: Starlix (nateglinide), Prandin (repaglinide), and Glufast (mitiglinide).

Currently, there is minimal direct evidence connecting meglitinides to changes in PD risk. Rhee et al. [183] and Xie et al. [184] found an increase PD risk associated with the use of glinides, whereas Sunnarborg et al. reported no significant change in PD risk.

The absence of specific studies on meglitinides and Parkinson’s disease highlights a gap in research, and as the research of diabetes and neurodegeneration continues to evolve, future studies may shed more light on the broader impacts of various diabetes treatment in Parkinson’s disease.

### 2.7. Alpha-Glucosidase Inhibitors (AGIs)

**Figure 11:**
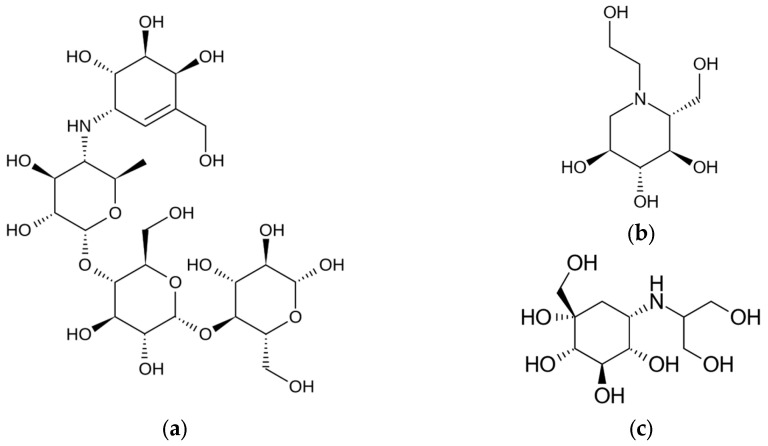
The chemical structure of acarbose (**a**), miglitol (**b**), and voglibose (**c**). Reproduced from Wikimedia Commons, https://commons.wikimedia.org/wiki/File:Acarbose.svg; https://commons.wikimedia.org/wiki/File:Miglitol.svg; https://commons.wikimedia.org/wiki/File:Voglibose.svg. Accessed 30 December 2024.

AGIs are a class of oral antidiabetic drugs primarily used to manage T2D by slowing down the digestion of carbohydrates, thereby reducing postprandial blood glucose levels (Figure 11 and Table 7). They are prescribed when glycemic control is not achieved with diet and physical activity alone.

While AGIs are not directly targeted at the dopaminergic system, their impact on glucose metabolism, gut–brain signaling, and oxidative stress may influence Parkinson’s disease’s pathogenesis. As hyperglycemia and insulin resistance have been implicated in the pathogenesis of Parkinson’s disease, by improving glycemic control, AGIs may indirectly exert neuroprotective effects. They may reduce oxidative stress by stabilizing blood glucose levels, potentially mitigating one of the pathways of neuronal damage in PD [185,186]. Some studies have suggested that AGIs might have anti-inflammatory effects, which could be beneficial in reducing neuroinflammation associated with Parkinson’s disease [187]. The gut–brain axis is increasingly recognized as an important factor in PD. AGIs can alter gut microbiota composition, which might influence neuroinflammatory and neurodegenerative processes [188].

AGIs are effective and generally safe, but their use requires careful consideration of potential side effects and patient-specific factors. Gastrointestinal side effects, such as bloating and diarrhea, limit their use. While AGIs offer an intriguing avenue for research in Parkinson’s disease, their use in this context is not documented. Continued exploration into their effects on metabolic pathways, oxidative stress, and inflammation could uncover new therapeutic strategies for managing PD.

### 2.8. T2D Drugs Which Are/Were in Clinical Trials Repurposing for the Treatment of Parkinson’s Disease

Several clinical trials have explored the repurposing of T2D medications for the treatment of PD. This approach is based on their shared pathological mechanisms. Below is a comprehensive overview of key clinical trials that have investigated or are currently investigating T2D drugs as potential therapies for PD up to October 2024 (the source is ClinicalTrials.gov https://clinicaltrials.gov/).

***1.***  ***Exenatide***

***NCT01174810 (2010–2013)—NCT01174810 (2010–2013)—****a randomized, double-blind, placebo-controlled, single-center, 60-week trial of Exenatide once weekly for the treatment of moderate severity Parkinson’s disease—completed, results published* [116]. *The results showed that Exenatide was well tolerated and improved motor and cognitive functions in PD patients.*

***NCT01971242 (2014–2016)—****a randomized, double-blind, placebo-controlled, single-center, 60-week trial of Exenatide once weekly for the treatment of moderate severity Parkinson’s disease—completed, results published* [118]. *Exenatide significantly reduced the deterioration of motor symptoms in patients with PD and improved nonmotor symptoms, cognition, and quality of life.*

***NCT03456687 (2018–2021)—*** *effects of Exenatide on motor function and the brain—completed.*

***NCT04232969 (2020-recruiting)—*** *a randomized, double-blind, parallel-group, placebo-controlled, phase 3 trial of Exenatide once weekly over 2 years as a potential disease modifying treatment for Parkinson’s disease.*

***NCT04305002 (2020-unknown)*** *—effect of Exenatide on disease progression in early Parkinson’s disease.*

***NCT04269642 (2020-unknown)*** *—phase IIa study to evaluate the efficacy and safety of subcutaneous sustained release (SR)-Exenatide (pt320) in patients with early Parkinson’s disease.*

***NCT04154072 (2020–2023)*** *—multi-center, randomized, double-blind, placebo-controlled study to evaluate the efficacy, safety, and tolerability of 36 weeks of treatment with NLY01 (a pegylated form of Exenatide) in early-stage Parkinson’s disease.*

***2.***  ***Liraglutide***

***NCT02953665 (2017–2022)****—a phase II, randomized, double-blind, placebo-controlled trial of liraglutide in Parkinson’s disease—completed, results published in* [121]. *The results proved liraglutide to be safe and enhances key aspects of PD, such as non-motor symptoms, overall mobility, daily living activities, and quality of life.*

***3.***  ***Pioglitazone***

***NCT01280123 (2011–2014)—****a multi-center, double-blind, placebo-controlled phase II study of Pioglitazone in early Parkinson’s disease—completed, results published in* [189]. *The study does not prove to modify progression in early PD.*

***4.***  ***Semaglutide***

***NCT03659682 (2019-recruiting)—*** *a single-center, double-blind, placebo-controlled study of semaglutide in idiopathic PD.*

***5.***  ***Lixisenatide***

***NCT03439943 (2018–2021)*** *—multi-center, randomized, placebo-controlled, double-blind, parallel arm proof-of-concept trial of lixisenatide in patients with early Parkinson’s disease—completed.*

***6.***  ***Metformin***

***NCT03685357 (2018-unknown)—****pilot study, investigation of the possible correlation between idiopathic Parkinson’s disease and diabetes mellitus in Egyptian elderly patients. Findings reported in study* [190] *indicated that individuals with diabetes exhibited elevated REM sleep behavior disorders scores, suggesting that these disturbances may serve as a premotor feature of PD.*

***NCT05781711 (2023-recruiting)—*** *clinical study to evaluate the possible efficacy of metformin in patients with Parkinson’s disease.*

## 3. Conclusions

The limitations of current Parkinson’s disease therapies emphasize the urgent need for disease-modifying treatments. The convergence of pathological pathways of T2D and PD suggests that repurposing antidiabetic medications offers a promising therapeutic avenue.

Metformin exhibits significant potential as a therapeutic agent in PD due to its diverse mechanisms of action that target key pathological features of PD. Metformin exhibits multiple mechanisms that may inhibit ferroptosis: by activating AMPK, metformin reduces lipid synthesis, particularly of PUFAs vulnerable to peroxidation, and enhances antioxidant defenses through the upregulation of GPx4 and GSH levels, modulates iron homeostasis, reducing iron-induced oxidative stress. However, conflicting evidence from various studies underscores the complexity of metformin’s effects in neuronal systems. While some research supports its neuroprotective roles, other findings suggest potential exacerbation of neuronal damage under certain conditions.

Incretin mimetics, including GLP-a and GIP receptor agonists, represent a promising class of therapeutics for the treatment of Parkinson’s disease. They exhibit promising potential in inhibiting ferroptosis through multiple mechanisms: the reduction of iron overload, antioxidant enhancement, regulation of ferroptosis markers, and reduction of ER stress. The interplay between metabolic regulation and neuronal survival underscores the importance of further research in this area. Detailed investigations into how incretin mimetics influence ferroptosis at the molecular level in neuronal cells are still needed; ferroptosis-specific biomarkers need to be identified and validated in PD patients to monitor treatment effects; and large-scale, long-term studies assessing the efficacy of incretins mimetics on ferroptosis inhibition and PD progression need to be done.

Dipeptidyl peptidase-4 inhibitors have shown neuroprotective effects in PD models. Although direct evidence linking DPP-4 inhibitors to ferroptosis inhibition is limited, their ability to reduce oxidative stress and inflammation suggests potential indirect effects on ferroptotic pathways. Additionally, their ability to enhance incretin levels, which have neuroprotective effects, may further contribute to their therapeutic potential.

By reducing lipid peroxidation, enhancing antioxidant defenses, modulating iron homeostasis, and attenuating inflammation, TZDs like rosiglitazone and pioglitazone may protect dopaminergic neurons from ferroptotic cell death. While preclinical studies are promising, inconsistent clinical results and safety concerns highlight the need for further research.

Sodium-glucose cotransporter-2 inhibitors may indirectly inhibit ferroptosis by reducing oxidative and nitrosative stress, enhancing antioxidant defenses, and modulating lipid peroxidation. While preliminary evidence suggests potential benefits in the context of Parkinson’s disease, direct evidence remains limited and inconsistent. Conflicting epidemiological data on the risk of PD associated with SGTT2i use highlight the need for comprehensive research to clarify their role.

Both meglitinides and alpha-glucosidase inhibitors currently lack direct evidence linking them to the modulation of ferroptosis in the context of Parkinson’s disease. Meglitinides primarily influence glucose metabolism without significantly impacting oxidative stress, inflammation, or iron handling—key factors in ferroptosis. AGIs, while potentially reducing oxidative stress and inflammation through improved glycemic control and gut microbiota modulation, have not been direct studied in relation to ferroptosis of PD.

The repurposing of antidiabetic medications for neurodegenerative diseases is an area of active research. Future research should adopt a multidimensional approach, integrating detailed molecular studies with robust clinical trials to bridge the translational gap. As of now, their use in PD remains investigational and off-label, and it is imperative that future studies provide more definitive insights into their potential roles.

## Figures and Tables

**Table 1:** Neuroprotective effects of metformin.

Key Mechanism	Details
**Reduction of Oxidative Stress**	**Decrease in ROS Production:** Metformin inhibits reverse electron flux through mitochondrial complex I, reducing ROS production and preventing oxidative stress and cell death [50]. **Enhancement of Antioxidant Systems:** Increases levels of glutathione (GSH) and superoxide dismutase (SOD), enhancing the cell’s ability to neutralize ROS [56,58]. **Scavenging Free Radicals:** Directly scavenges hydroxyl free radicals and indirectly decreases ROS through NADPH oxidase or the mitochondrial respiratory chain [53,54]. **Induction of MnSOD:** Promotes manganese-dependent superoxide dismutase (MnSOD) and mitochondrial biogenesis, reducing mitochondrial ROS [51].
**Enhancement of Autophagy and Protein Homeostasis**	**Activation of Autophagy Pathways:** Enhances autophagy, promoting the elimination of α-syn aggregates, which are neurotoxic in PD [65,66,67]. **Reduction of α-syn Aggregation:** Decreases α-syn aggregation and dopaminergic neuron loss in PD models [65,67]. **Regulation of Protein Phosphorylation:** Reduces levels of phosphorylated α-syn at serine 129 by activating protein phosphatase 2A (PP2A) via AMPK-dependent and independent pathways [63,64].
**Energy Metabolism and Mitochondrial Function**	**AMPK Activation:** Activates AMPK, promoting metabolic balance and neuronal survival. AMPK activation leads to inhibition of lipid biosynthesis and maintenance of ATP levels, crucial for neuronal health [36,37,38,39]. **Mitochondrial Protection:** Causes mild inhibition of mitochondrial complex I, maintaining ATP production and reducing energy stress. Promotes mitochondrial biogenesis, enhancing mitochondrial function and resilience [30,31,32,33,34,51].
**Anti-Inflammatory Effects**	**Reduction of Inflammation:** Regulates changes in astrocytes and microglia, reducing neuroinflammation and protecting dopaminergic neurons [73].
**Protection of Dopaminergic Neurons and Improvement in PD Models**	**Protective Effects in PD Models:** Reduces dopaminergic neuron loss and α-syn accumulation in various PD models [65,67,69]. **Enhancement of Motor Functions:** Improves motor performance in MPTP-induced PD mice by restoring dopamine levels and reducing pathological markers [65,68]. **Reduction of Neurotoxic Aggregates:** Prevents MGO-induced α-syn oligomerization, mitigating neurodegeneration [19,66,67].
**Modulation of Signaling Pathways**	**Nrf2 Pathway Activation:** Activates the Nrf2 signaling pathway, enhancing cellular antioxidant defenses and reducing oxidative damage [56]. **AMP-Dependent Pathways:** Utilizes alternative AMPK activation routes, such as the lysosomal pathway, stabilizing cellular energy homeostasis and resilience against stress [40].
**Miscellaneous Neuroprotective Mechanisms**	**Indirect Effects on Insulin Sensitivity:** Enhances insulin sensitivity in peripheral tissues, potentially supporting overall neuronal health through metabolic regulation [25,26]. **Scavenging Advanced Glycation End-Products (AGEs):** Acts as a scavenger of methylglyoxal (MGO) and may prevent the accumulation of AGEs, reducing neurotoxicity and protein dysfunction [19,66,67,68].
**Inhibition of Ferroptosis**	**Modulation of Ferroptosis Markers:** Increases GPx4 and suppresses ACSL4 levels, preventing lipid peroxidation and ferroptosis [56,70,71]. **Regulation of Iron Homeostasis:** Upregulates ferroportin (FPN) expression, reducing iron overload and enhancing iron detoxification. Reduces ferritin heavy chain expression, improving iron homeostasis [59]. **Enhancement of Antioxidant Defenses:** Elevates GSH levels and SOD activity, strengthening defenses against ferroptosis [58,69]. **Inhibition of Lipid Peroxidation:** Decreases malondialdehyde (MDA) and 4-hydroxynonenal (4-HNE) levels, reducing markers of lipid peroxidation [55,67].

**Table 2:** FDA approved GIP and GLP-1 receptor agonists.

Generic Name/Trade Name	Indication	Approval Year
Exenatide (Byetta^®^)	T2D	2005
Liraglutide (Victoza^®^)	T2D	2010
Dulaglutide (Trulicity^®^)	T2D	2014
Liraglutide (Saxenda^®^)	Obesity/overweight	2014
Lixisenatide (Adlyxin^®^)	T2D	2016
Liraglutide + insulin degludec (Xultophy^®^)	T2D	2016
Lixisenatide + insulin glargine (Soliqua^®^)	T2D	2016
Exenatide extended release (Bydureon^®^)	T2D	2017
Semaglutide injection (Ozempic^®^)	T2D	2017
Semaglutide tablets (Rybelsus^®^)	T2D	2019
Semaglutide (Wegovy^®^)	Obesity/overweight	2021
Tirzepatide (Mounjaro^®^) (dual GLP-1/GIP receptor agonist)	T2D	2022
Tirzepatide (Zepbound^®^) (dual GLP-1/GIP receptor agonist)	Obesity/overweight	2023

**Table 3:** Neuroprotective effects of incretin mimetics.

Key Effects	Details
**Neuroprotective Efficacy and** **Neuronal Protection**	**Synaptic Protection and Synaptogenesis:** Protects synapses and promotes the formation of new synapses, enhancing neural connectivity [99]. **Enhancement of Synaptic Plasticity:** Improves the adaptability of synapses, facilitating learning and memory consolidation [99]. **Rescue of Cognitive Decline:** Prevents or reverses deterioration in cognitive functions [90,94]. **Regulation of Glial Cell Functions:** Modulates microglia and astrocyte activation, reducing neuroinflammation [112,113,114]. **Prevention of Ca^2^⁺ Overload:** Protects neurons from calcium-induced toxicity [99]. **Protection of Nigrostriatal Neurons:** Safeguards dopaminergic neurons in the nigrostriatal pathway [100,101,102]. **Dopamine Replenishing:** Restores dopamine levels in the brain, improving motor functions [100,101,102].
**Stress and Inflammation Reduction, Anti-Apoptotic Effects**	**Suppression of ER Stress:** Reduces endoplasmic reticulum stress, preventing protein misfolding and aggregation [104,107]. **Anti-Inflammatory Effects:** Decreases neuroinflammation by reducing pro-inflammatory cytokines [87,112,113,114,126]. **Protection from External Oxidative Stress:** Mitigates damage caused by oxidative agents [99,100,101,102,104,107]. **Anti-Apoptotic Activities:** Prevents programmed cell death in neurons [112,113,114].
**Mitochondrial Protection**	**Mitigation of Mitochondrial Dysfunction:** Improves mitochondrial integrity and function, ensuring efficient energy production [110]. **Recovery of Mitochondrial Function:** Restores normal mitochondrial operations, preventing energy deficits [110]. **Reduction of Superoxide Formation:** Decreases superoxide radical production, preventing oxidative mitochondrial damage [126,127,128,129].
**Ferroptosis Inhibition**	**Reduction of Iron Overload:** Decreases iron accumulation in the brain and other tissues, preventing iron-induced oxidative damage [94,107,125,126,127,128,129,131]. **Modulation of Ferroptosis Markers:** Increases GPx4 and SLC7A11 expression, and decreases ACSL4 levels, thereby inhibiting ferroptosis [110]. **Enhancement of Antioxidant Defenses:** Elevates GSH and SOD levels, strengthening cellular defenses against ferroptosis [107,108,126,127,128,129,130,131]. **Activation of Antioxidant Signaling Pathways:** Activates the Nrf2/HO-1 pathway, enhancing overall antioxidant capacity [107,126]. **Regulation of Antioxidant Proteins:** Increases Bcl-2 and Bcl-xL, which scavenge free radicals and inhibit superoxide anion formation [111]. **Reduction of Lipid Peroxidation:** Lowers MDA and HNE levels, reducing markers of lipid peroxidation [99,108,131].
**Improvement in Motor and Cognitive Functions**	**Enhanced Motor Performance:** Improves motor functions in PD models and patients through dopamine replenishing and neuronal protection [115,116,117,118,119,120,121]. **Cognitive Function Improvement:** Enhances cognitive capabilities and reduces cognitive decline through synaptic and neuronal support [90,94,99,120,126,127,128,129,130,131].
**Iron Metabolism Regulation**	**Reduction of Iron Overload in Tissues:** Lowers iron levels in the liver and brain, preventing iron-induced oxidative stress [94,107,131]. **Upregulation of Iron Exporters:** Increases ferroportin (FPN) expression and decreases transferrin receptor (TfR1) expression, improving iron homeostasis and reducing iron import [107,128,129,130,131].
**Neurogenesis Enhancement**	**Promotion of Adult Neurogenesis:** Encourages the formation of new neurons in the adult brain, particularly in the hippocampus [90,94]. **Increase in Neuronal Progenitor Cells:** Boosts the population of neuronal progenitor cells, aiding in neuronal regeneration [94].
**Oxidative Stress Reduction**	**Scavenging of Free Radicals:** Directly scavenges hydroxyl radicals and indirectly reduces ROS production through DPP-4 and mitochondrial pathways [52,53,55,87]. **Upregulation of Antioxidant Enzymes:** Increases the expression of MnSOD, GPx4, and other antioxidant enzymes, enhancing the cellular capacity to neutralize ROS [107,108,126,127,128,129,130,131].
**Protection against Dopaminergic Neuron Loss**	**Reduction of α-Synuclein Aggregation:** Prevents the accumulation of toxic α-syn oligomers, mitigating their neurotoxic effects [99,112,113,114,131]. **Protection from Dopaminergic Neuron Degeneration:** Safeguards neurons that produce dopamine, reducing neuron loss and improving motor functions [100,101,120,121,122,123,124,125,126,127,128,129,130,131].

**Table 4:** Marketed approved gliptins as monotherapy and in combination therapies ^1^.

Generic Name/Trade Name	Approved by	Approval Year
Sitagliptin (Januvia)	FDA	2006
Vildagliptin (Galvus)	EU	2007
Saxagliptin (Onglyza)	FDA	2009
Linagliptin (Tradjenta)	FDA	2011
Gemigliptin (Zemiglo)	Korea	2012
Anagliptin (Suiny)	Japan	2012
Teneligliptin (Tenelia)	Japan	2012
Alogliptin (Nesina)	FDA	2013
Trelagliptin (Zafatek/Wedica)	Japan	2015
Omarigliptin (Marizev)	Japan	2015
Sitagliptin (Zituvio)	FDA	2023
Evogliptin (Suganon/Evodine)	Korea	-
Gosogliptin (Saterex)	Rusia	-

^1^ (From Wikipedia, accessed 23 October 2024).

**Table 5:** Neuroprotective effects of DPP-4 inhibitors.

Key Mechanisms	Details
**Neuroprotective Efficacy and Neuronal Protection**	**Promotes neuronal growth and survival** [146] **Enhances neurotrophic factors, supporting neuronal health and function** [146] **Prevents Dopamine Reduction and Dopaminergic Neuron Loss:** Protects dopaminergic neurons from degeneration, preserving dopamine levels in the brain [135,145,146].
**Stress and Inflammation Reduction, Anti-Apoptotic Effects**	**Downregulation of ER Stress Markers:** Reduces ER stress by downregulating CHOP, TRIB3, and activating ATF-4, thereby preventing protein misfolding and apoptosis [141,142,143]. **Suppress Inflammatory Pathways:** Inhibits inflammatory signaling pathways, reducing the production of pro-inflammatory cytokines [135,145]. **Prevention of Apoptosis:** Prevents programmed cell death in neurons by reducing oxidative and nitrosative stress [135,145,146].
**Mitochondrial Protection**	**Restoring Mitochondrial Complex I Activity and Bcl-2 Levels:** Enhances mitochondrial function and integrity, maintaining ATP synthesis and preventing energy deficits [145].
**Ferroptosis Inhibition**	**Improvement of Iron Metabolism:** Regulates iron-related proteins such as TfR1 and FPN1, reducing iron overload and enhancing iron detoxification [141,143]. **Reduction of Oxidative and Nitrosative Stress:** Decreases iNOS transcription and MPO activity, inhibiting ROS and RNS production [144,145]. **Reduction of lipid peroxidation**: Decreases TBARS levels [144] **Activation of Antioxidant Signaling Pathways (Nrf2):** Enhances antioxidant defenses and reduces lipid peroxidation [135,145].

**Table 6:** Comparison of incretin mimetics and DPP-4 inhibitors in combating Parkinson’s disease.

Aspect	Incretin Mimetics	DPP-4 Inhibitors
**Mechanism of Action**	Directly activate GLP-1receptors enhancing insulin secretion and neuroprotection	Inhibit DPP-4 enzyme, increasing endogenous GLP-1 and GIP levels
**Neuroprotective Efficacy**	Strong neuroprotective effects through direct activation of GLP-1Rs, promoting neurogenesis, synaptic plasticity, and reducing oxidative stress and inflammation	Moderate neuroprotective effects; primarily reduce inflammation and apoptosis indirectly by increasing GLP-1 levels
**BBB Permeability**	Some (e.g., Exenatide, lixisenatide) can cross the BBB, allowing direct central nervous system effects	Generally limited BBB permeability, restricting central effects
**Impact on Ferroptosis-related Pathways**	Potential impact on ferroptosis through reducing oxidative stress, improving mitochondrial function, and enhancing antioxidant pathways (e.g., Nfr2/HO-1)	Limited direct evidence on ferroptosis; potential indirect effects through reducing oxidative stress and inflammation
**Clinical Evidence in PD**	Demonstrated benefits in motor and cognitive functions in clinical trial; potential disease-modifying effects	Some positive effects in preclinical models; clinical evidence less robust compared to GLP-1RAs
**Combination Therapy Potential**	Can be combined with other therapies for enhanced neuroprotective effects	Potential to enhance GLP-1 activity and complement other PD treatments

**Table 7:** FDA-approved AGIs.

Generic Name/Trade Name	Indication	Approval Year
Acarbose (Precose^®^)	T2D	1995
Miglitol (Glyset^®^)	T2D	1996
Voglibose (Voglib^®^)	T2D	Only in Japan
